# Progressive von Mises–Fisher Filtering Using Isotropic Sample Sets for Nonlinear Hyperspherical Estimation †

**DOI:** 10.3390/s21092991

**Published:** 2021-04-24

**Authors:** Kailai Li, Florian Pfaff, Uwe D. Hanebeck

**Affiliations:** Intelligent Sensor-Actuator-Systems Laboratory (ISAS), Institute for Anthropomatics and Robotics, Karlsruhe Institute of Technology (KIT), 76131 Karlsruhe, Germany; florian.pfaff@kit.edu (F.P.); uwe.hanebeck@kit.edu (U.D.H.)

**Keywords:** sensor fusion, recursive Bayesian estimation, directional statistics, unscented transform, nonlinear hyperspherical filtering

## Abstract

In this work, we present a novel scheme for nonlinear hyperspherical estimation using the von Mises–Fisher distribution. Deterministic sample sets with an isotropic layout are exploited for the efficient and informative representation of the underlying distribution in a geometrically adaptive manner. The proposed deterministic sampling approach allows manually configurable sample sizes, considerably enhancing the filtering performance under strong nonlinearity. Furthermore, the progressive paradigm is applied to the fusing of measurements of non-identity models in conjunction with the isotropic sample sets. We evaluate the proposed filtering scheme in a nonlinear spherical tracking scenario based on simulations. Numerical results show the evidently superior performance of the proposed scheme over state-of-the-art von Mises–Fisher filters and the particle filter.

## 1. Introduction

The use of inferences on (hyper-)spherical states is ubiquitous in a large variety of application scenarios, such as protein structure prediction [1], rigid-body motion estimation [2,3], remote sensing [4], omnidirectional robotic perception [5,6] and scene segmentation and understanding [7,8]. In most of these tasks, quantifying uncertainties in hyperspherical domains is crucial (for brevity, the word “hypersphere” is used to denote spheres of any dimension in unspecified cases throughout the paper) . Therefore, the von Mises–Fisher distribution [9,10] has become a popular probabilistic model defined on the unit hypersphere $S^{d-1}=\left\{\underline{x}\in R^{d}:∥\underline{x}∥=1\right\}$.

The recursive estimation of hyperspherical random variables using the von Mises–Fisher distribution is nontrivial due to its nonlinear and periodic nature on directional manifolds [10]. Samples generated from the underlying distribution are typically employed to propagate estimates through system dynamics or to evaluate the likelihoods given certain measurements. In [11,12], rejection sampling-based approaches were proposed to generate random samples for von Mises–Fisher distributions in arbitrary dimensions with an unbounded runtime. A deterministic runtime without resorting to rejection schemes is achievable, although only for specific numbers of dimensions; e.g., using the methods proposed in [13] on the unit sphere $S^{2}$ or that given in [14] for odd numbers of dimensions.

Although a random sampling-based von Mises–Fisher filter is effective for nonlinear hyperspherical estimation, it cannot deliver reproducible results and may lack runtime efficiency (especially under strong nonlinearities or in high-dimensional state spaces). Therefore, deterministic sample sets are desired for an efficient and accurate representation of the underlying distribution. Reminiscent of the unscented Kalman filter (UKF) for linear domains, the so-called unscented von Mises–Fisher filter (UvMFF) was proposed in [15] on unit hyperspheres $S^{d-1}\in R^{d}$. Following the idea of the unscented transform (UT), 2d−1 deterministic samples are drawn in a way that preserves the mean resultant vector of the underlying von Mises–Fisher distribution. Compared with confining a UKF to the manifold structure, this approach delivers superior performance for nonlinear hyperspherical tracking.

There remains considerable space for improvement for state-of-the-art von Mises–Fisher filtering schemes in the area of high-performance hyperspherical estimation. The deterministic sampling method used in the current UvMFF [15] only allows fixed numbers of hyperspherical samples (i.e., 2d−1 samples on $S^{d-1}$) in accordance with the unscented transform. Moreover, the current UvMFF only allows identity measurement models with the measurement still confined to hyperspheres, and the measurement noise must be von Mises–Fisher-distributed. Thus, its practical deployment to arbitrary sensor modalities requires reapproximating the measurement model and the noise term [16], leading to additional preprocessing and errors. For arbitrary measurement models, directly reweighting the samples and fitting a posterior von Mises–Fisher to them is theoretically feasible. However, a limited number of deterministic samples is prone to degeneration, which is particularly risky under strong nonlinearities or with peaky likelihood functions. Therefore, it is important to enable deterministic sample sets of flexible sizes to better represent the underlying distribution while satisfying the condition of the unscented transform.

Generating deterministic sample sets of configurable sizes for continuous distributions was originally investigated in Euclidean spaces. In [17], deterministic samples were generated from a multivariate Gaussian distribution by minimizing the statistical divergence between its supporting Dirac mixture and the underlying continuous densities. For this, the Cramér–von Mises distance was generalized to the multivariate case based on the so-called localized cumulative distribution (LCD) to quantify the statistical divergence. This Dirac mixture approximation (DMA)-based method was further improved in [18] for better efficiency and extended in [19] for Gaussian mixtures.

In the context of recursive estimation based on distributions from directional statistics [10], deterministic samples are typically drawn by preserving moments up to a certain order. In [20], five deterministic samples were generated for distributions on circular domains; e.g., for the wrapped normal or the von Mises distribution [21]. For this, a sample set was scaled to match the first and the second trigonometric moments of the distribution. Sample sets for different scaling factors can then be merged to a larger set via superposition. In [22], a DMA-like sampling scheme was proposed to generate arbitrary numbers of deterministic samples while preserving the circular moments via optimization. In [23], deterministic samples were drawn from typical circular distributions via optimal quadratic quantification based on the Voronoi cells. For unit hyperspheres, major efforts have been dedicated to the Bingham distribution, where the basic UT-based sampling scheme in [24] (2d−1 samples as for $S^{d-1}\in R^{d}$) was extended for arbitrary sample sizes, first in the principal directions [25] and then for the entire hyperspherical domain [26]. The sampling paradigm was DMA-based, with an on-manifold optimizer minimizing the statistical divergence of the samples to the underlying distribution under the moment constraints up to the second order. Based on this, improved filtering performance has been shown for quaternion-based orientation estimation.

Although non-identity measurement models can be handled by enlarging the sizes of deterministic samples in the update step, considerably large sample sizes are still desired in the face of degeneration issues (e.g., due to strong nonlinearities or peaky likelihoods). Thus, there remains the necessity to improve sample utilization. In [27], a novel progressive update scheme was proposed for nonlinear Gaussian filtering by decomposing the likelihood into a product of likelihoods with exponents adaptively determined by confining sample weight ratios within a pre-given threshold. Consequently, deterministic samples of small sizes are less likely to degenerate and become more deployable for nonlinear estimation. Similar schemes have also been proposed for estimating angular systems [28] and Bingham-based hyperspherical filtering [29] with non-identity measurement models.

To date, there exists no flexible deterministic sampling scheme for von Mises–Fisher distributions. Existing optimization-based paradigms may have undesirable properties, such as local minima or a deteriorated runtime, for large sample sizes, which prohibit their deployment to online estimation tasks. Furthermore, no sample-efficient method is available for von Mises–Fisher filtering with non-identity measurement models.

In consideration of the state-of-the-art approaches above, we propose a novel algorithm for von Mises–Fisher distributions in arbitrary dimensions to obtain deterministic sample sets with manually configurable sizes. Based on hyperspherical geometries, samples are drawn coherently to the isotropic dispersion of the underlying distribution without optimization while satisfying the requirement of the unscented transform. Moreover, a novel progressive update scheme is developed in conjunction with the proposed sampling approach for nonlinear von Mises–Fisher filtering. Furthermore, an extensive evaluation of nonlinear spherical estimation is provided. Compared with existing von Mises–Fisher filtering schemes and the particle filter, the proposed progressive von Mises–Fisher filter using isotropic sample sets delivers superior performance with regard to tracking accuracy, runtime and memory efficiency.

The remainder of the paper is structured as follows. Preliminaries for the von Mises–Fisher distributions and the corresponding hyperspherical geometry are given in Section 2. Based on this, the novel isotropic deterministic sampling scheme is introduced in Section 3. In Section 4, the proposed progressive deterministic update for von Mises–Fisher filtering is provided, followed by a simulation-based benchmark of nonlinear spherical tracking in Section 5. The work is concluded in Section 6.

## 2. Preliminaries

### 2.1. General Conventions of Notations

We use underlined lowercase variables $\underline{x}\in R^{d}$ to denote vectors. Random variables are denoted by lowercase boldface letters $\underline{x}$. Uppercase boldface letters B are used to denote matrices. $S^{d-1}\subset R^{d}$ denotes the unit (d−1)-sphere embedded in the *d*-dimensional Euclidean space. In the context of recursive Bayesian estimation, we denote the posterior density of the state at time step *t*, which relates to all measurements up to time step *t*, by $f_{t}^{e}$. $f_{t+1}^{p}$ is used for the predicted density of the state at time step t+1 with regard to all measurements up to *t*. The rest of the symbols used are explained in the course of the following pages.

### 2.2. The von Mises–Fisher Distribution

Defined on the unit hypersphere $S^{d-1}\subset R^{d}$, the von Mises–Fisher distribution $\underline{x}∼\mathrm{VMF}\left(\underline{\nu },\kappa \right)$ is parameterized by the mode location $\underline{\nu }\in S^{d-1}\subset R^{d}$ and the concentration parameter κ≥0. Its probability density function is given in the form

(1) $$f_{\mathrm{vMF}}\left(\underline{x}\right)=N_{d}\left(\kappa \right)\cdot \exp\left(\kappa \;{\underline{\nu }}^{⊤}\underline{x}\right),\;\underline{x}\in S^{d-1}\;,$$

with the normalization constant

(2) $$N_{d}\left(\kappa \right)={\left(\int_{S^{d-1}}\exp\left(\kappa \;{\underline{\nu }}^{⊤}\underline{x}\right)\;d\underline{x}\right)}^{-1}=\frac{\kappa^{d/2-1}}{{\left(2\pi \right)}^{d/2}\;I_{d/2-1}\left(\kappa \right)}$$

depending on the concentration κ and the dimension *d*. $I_{d/2-1}\left(\kappa \right)$ denotes the modified Bessel function of the first kind and order d/2−1. Note that the von Mises–Fisher distribution quantifies uncertainties using an arc length-based metric, which is coherent to the hyperspherical manifold structure. The distribution is unimodal and exhibits an isotropic dispersion. By generalizing the trigonometric moment from the circular to the hyperspherical domain, we obtain the *mean resultant vector* of the von Mises–Fisher distribution as follows

$$\underline{\alpha }=E\left(\underline{x}\right)=\int_{S^{d-1}}\underline{x}f_{\mathrm{vMF}}\left(\underline{x}\right)\;d\underline{x}=A_{d}\left(\kappa \right)\;\underline{\nu }\;,\;\mathrm{with}\;A_{d}\left(\kappa \right)=\frac{I_{d/2}\left(\kappa \right)}{I_{d/2-1}\left(\kappa \right)}$$

denoting the ratio of two Bessel functions. Therefore, the mean resultant vector is essentially a re-scaled hyperspherical mean $\underline{\nu }$, with a length of $∥\underline{\alpha }∥=A_{d}\left(\kappa \right)$. Given a set of weighted samples ${\left\{\left({\underline{x}}_{i},\omega_{i}\right)\right\}}_{i=1}^{n}\subset S^{d-1}$ with the weights $\sum_{i=1}^{n}\omega_{i}=1$, a von Mises–Fisher distribution can be fitted to the sample mean $\hat{\underline{\alpha }}=\sum_{i=1}^{n}\omega_{i}\;{\underline{x}}_{i}$ via

(3) $$\hat{\underline{\nu }}=\hat{\underline{\alpha }}/\left|\left|\hat{\underline{\alpha }}\right|\right|\;\;\mathrm{and}\;\;\hat{\kappa }=A_{d}^{-1}\left(\hat{\underline{\alpha }}\right)\;.$$

To obtain the concentration $\hat{\kappa }$, one needs to solve the inverse of the Bessel function ratio in Equation (2), which can be efficiently obtained using the algorithm introduced in [30]. Moment matching to the mean resultant vector has been proven to be equivalent to maximum likelihood estimation (MLE) [31] (Section A.1) for the von Mises–Fisher distribution. Moreover, this also guarantees minimal information loss (quantified by the Kullback–Leibler divergence) when fitting a von Mises–Fisher to an arbitrary distribution [32] for stochastic filtering.

### 2.3. Geometric Structure of Hyperspherical Manifolds

The von Mises–Fisher distribution quantifies hyperspherical uncertainty in relation to the geodesic curve length on the manifold to the mode. To establish the proposed deterministic sampling scheme, we first investigate the hyperspherical domain from the perspective of Riemannian geometry [33]. Any point $\underline{x}\in S^{d-1}$ can be mapped to the tangent space at $\underline{\nu }\in S^{d-1}$ via the logarithm map

$$\tilde{\underline{x}}={\mathrm{Log}}_{\underline{\nu }}\left(\underline{x}\right)=\left(\underline{x}-\cos\left(\gamma \right)\;\underline{\nu }\right)\frac{\gamma }{\sin(\gamma )}\in T_{\underline{\nu }}S^{d-1}\;,\mathrm{with}\;\gamma =\arccos\left({\underline{\nu }}^{⊤}\underline{x}\right)\;,$$

while preserving its geodesic distance to $\underline{\nu }$; i.e., $|\gamma |=∥{\mathrm{Log}}_{\underline{\nu }}\left(\underline{x}\right)∥$. Inversely, any point $\tilde{\underline{x}}\in T_{\underline{\nu }}S^{d-1}$ can be retracted to the unit hypersphere via the exponential map

$$\underline{x}={\mathrm{Exp}}_{\underline{\nu }}\left(\tilde{\underline{x}}\right)=\cos\left(∥\tilde{\underline{x}}∥\right)\underline{\nu }+\frac{\sin\left(∥\tilde{\underline{x}}∥\right)}{∥\tilde{\underline{x}}∥}\;\tilde{\underline{x}}\in S^{d-1}\;.$$

When expressing logarithm-mapped points $\tilde{\underline{x}}\in T_{\underline{\nu }}S^{d-1}$ with regard to an orthonormal basis of the tangent space, their local coordinates ${\tilde{\underline{x}}}^{l}$ essentially form a (d−1)-ball of radius π—i.e., ${\tilde{\underline{x}}}^{l}\in B_{\pi }^{d-1}\subset R^{d-1}$—which is bounded by the hypersphere $S_{\pi }^{d-2}$ of radius π. To avoid ambiguities, we denote the logarithm and exponential maps defined for hyperspherical geometry above with capitalized first letters to distinguish them from the common logarithmic and exponential functions used in algebra [33].

## 3. Isotropic Deterministic Sampling

Considering the isotropic dispersion of the von Mises–Fisher distribution, we design a sample set layout with one sun sample at the mode surrounded by λ hyperspherical orbits of interval ζ. On each orbit, τ planet samples are placed (quasi-)equidistantly, thereby inducing a sample set $X\subset S^{d-1}$ of cardinality λτ+1. All samples are equally weighted. One has to determine the interval value ζ that ensures that the samples are confined to the mean resultant vector of the underlying distribution, thereby preserving the unscented von Mises–Fisher filtering paradigm. Based on the introduction in Section 2, details about deriving the orbit interval follow.

**Figure 1:**
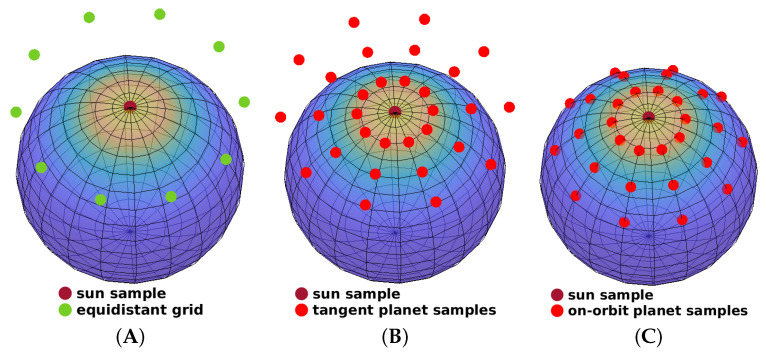
Illustration of isotropic deterministic sampling with (λ,τ)=(3,10) for a von Mises–Fisher distribution (κ=4) on $S^{2}$. (**A**) Equal partitioning in $T_{\underline{\nu }}S^{2}$ with regard to its local basis. (**B**) Scaling with the UT-preserving interval in $T_{\underline{\nu }}S^{2}$. (**C**) Exponential map from $T_{\underline{\nu }}S^{2}$ to $S^{2}$ for placing planet samples on hyperspherical orbits.

The proposed isotropic deterministic sampling scheme is detailed in Algorithm 1 and illustrated in Figure 1. Given a von Mises–Fisher distribution on $S^{d-1}$, we first place the sun sample at its mode (Algorithm 1, line 1). The tangent space at the mode, $T_{\underline{\nu }}S^{d-1}$, is bounded by the hypersphere $S_{\pi }^{d-2}$ with regard to its local basis $B_{\underline{\nu }}$ (Algorithm 1, line 2). To obtain τ planet samples on each hyperspherical orbit, we first generate equidistant grid points on the unit hypersphere $S^{d-2}$ using the equal area partitioning algorithm from [34] (Algorithm 1, line 3 and Figure 1A). Given the sampling configuration, the hyperspherical orbit interval ζ is then computed in accordance with the requirement of the unscented transform (Algorithm 1, line 4). Afterward, the obtained sample set ${\left\{{\tilde{\underline{x}}}_{s}^{l}\right\}}_{s=1}^{\tau }\subset S^{d-2}$ with regard to $\;B_{\underline{\nu }}$ is transformed into global coordinates scaled by each orbit radius rζ(r=1,…,λ) and undergoes the exponential map to land on the *r*-th orbit on $S^{d-1}$ (Algorithm 1, line 5–9, Figure 1B,C). In order to determine the orbit interval ζ that guarantees the unscented transform for filtering, we provide the following derivations.
**Algorithm 1:** Isotropic Deterministic Sampling
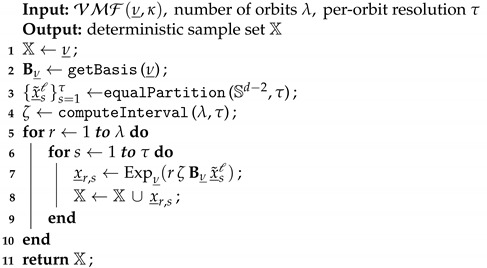


We map each point ${\tilde{\underline{x}}}_{s}^{l}$ generated by the equal area partitioning algorithm [34] with regard to $\;B_{\underline{\nu }}$ to $S^{d-1}$ according to Algorithm 1, line 7, and obtain

$${\underline{x}}_{r,s}={\mathrm{Exp}}_{\underline{\nu }}\left(r\;\zeta \;B_{\underline{\nu }}\;{\tilde{\underline{x}}}_{s}^{l}\right)=\cos\left(r\;\zeta \right)\;\underline{\nu }+\sin\left(r\;\zeta \right)\;B_{\underline{\nu }}\;{\tilde{\underline{x}}}_{s}^{l}\in S^{d-1}\;,$$

with ${\underline{x}}_{r,s}$ denoting the *s*-th planet sample on the *r*-th hyperspherical orbit. The bold letter $B_{\underline{\nu }}\in R^{d\times (d-1)}$ denotes the matrix transforming coordinates from the local basis $B_{\underline{\nu }}$ to the global one. Then, the hyperspherical mean of the whole sample set (including the sun sample) is

(4) $$\begin{array}{cc} \underline{\alpha }= & \frac{1}{\lambda \tau +1}\left(\underline{\nu }+\sum_{r=1}^{\lambda }\sum_{s=1}^{\tau }{\underline{x}}_{r,s}\right)\\ = & \frac{1}{\lambda \tau +1}\left(\underline{\nu }+\tau \sum_{r=1}^{\lambda }\cos\left(r\;\zeta \right)\;\underline{\nu }+\sum_{r=1}^{\lambda }\sum_{s=1}^{\tau }\sin\left(r\;\zeta \right)\;B_{\underline{\nu }}{\tilde{\underline{x}}}_{s}^{l}\right). \end{array}$$

For typical configurations of the equal area partitioning algorithm for unit hyperspheres [34], the sample set ${\left\{{\tilde{\underline{x}}}_{s}^{l}\right\}}_{s=1}^{\tau }$ is zero-centered. Therefore, the formula in Equation (4) can be further simplified as

$$\underline{\alpha }=\frac{1}{\lambda \tau +1}\left(1+\tau \sum_{r=1}^{\lambda }\cos\left(r\;\zeta \right)\right)\underline{\nu }\;.$$

By constraining the sample set mean to be identical to the mean resultant vector of the underlying distribution—i.e., $\underline{\alpha }=A_{d}\left(\kappa \right)\;\underline{\nu }$—the hyperspherical moment in Equation (3) is maintained, thereby satisfying the requirement of the unscented transform. Consequently, we have

$$\sum_{r=1}^{\lambda }\cos\left(r\;\zeta \right)=\frac{\left(\lambda \tau +1\right)A_{d}\left(\kappa \right)-1}{\tau }\;.$$

By exploiting Lagrange’s trigonometric identity [35] (Section 2.4.1.6), the finite series in the equation above can be further simplified, and we obtain

$$\frac{\sin\left((\lambda +0.5)\zeta \right)}{2\sin(0.5\;\zeta )}=\frac{\left(\lambda \tau +1\right)A_{d}\left(\kappa \right)-1}{\tau }+\frac{1}{2}\;.$$

The left-hand side fits the form of the (scaled) Dirichlet kernel [36] $D_{\lambda }\left(\zeta \right)$ and the desired orbit interval ζ is obtained by solving the equation

(5) $$D_{\lambda }\left(\zeta \right)=\frac{\left(\lambda \tau +1\right)A_{d}\left(\kappa \right)-1}{\tau }+\frac{1}{2}\;,\;\mathrm{with}\;D_{\lambda }\left(\zeta \right)=\frac{\sin\left((\lambda +0.5)\zeta \right)}{2\sin(0.5\;\zeta )}\;,\;\zeta \in \left[\;0,\frac{\pi }{\tau }\;\right]\;.$$

Note that Equation (5) does not have a closed-form solution. It is trivial to prove that the maximum of the Dirichlet kernel is obtained at ζ=0 with $D_{\lambda }^{\max}\left(\zeta \right)=D_{\lambda }\left(0\right)=\lambda +0.5$. Meanwhile, the constant on the right-hand side of Equation (5) is smaller than $D_{\lambda }^{\max}\left(\zeta \right)$ given the Bessel function ratio $A_{d}\left(\kappa \right)\in \left(0,1\right)$ for κ>0. Therefore, Equation (5) is solvable for ζ∈[0,π/τ].

### 3.1. Numerical Solution for Equation (5)

Instead of deploying a universal numerical solver (e.g., the function solve in Matlab) to solve Equation (5) as in our preceding work [37], we now provide a tailored Newton’s method with iterative steps of a closed form. For that, the first derivative of the Dirichlet kernel is provided as follows:

$$\begin{array}{cc} D_{\lambda }^{\prime }\left(\zeta \right) & =\frac{\left(\lambda +0.5\right)\cos\left((\lambda +0.5)\zeta \right)\sin\left(0.5\;\zeta \right)-0.5\;\sin\left((\lambda +0.5)\zeta \right)\cos\left(0.5\;\zeta \right)}{2{\left(\sin(0.5\;\zeta )\right)}^{2}}\\ \; & =0.5\;\left(\lambda +0.5\right)\cos\left((\lambda +0.5)\zeta \right)\csc\left(0.5\;\zeta \right)-0.5\;D_{\lambda }\left(\zeta \right)\cot\left(0.5\;\zeta \right)\;. \end{array}$$

Then, the (k+1)-th Newton step for updating $\zeta_{k}$ is given as

$$\zeta_{k+1}=\zeta_{k}-\frac{D_{\lambda }\left(\zeta_{k}\right)-\left(\left(\lambda \tau +1\right)A_{d}\left(\kappa \right)-1\right)/\tau -0.5}{0.5\;\left(\lambda +0.5\right)\cos\left(\left(\lambda +0.5\right)\zeta_{k}\right)\csc\left(0.5\;\zeta_{k}\right)-0.5\;D_{\lambda }\left(\zeta_{k}\right)\cot\left(0.5\;\zeta_{k}\right)}\;.$$

To initialize ζ of the Newton’s method, we perform a linear interpolation between 0 and the first non-negative zero of the Dirichlet kernel, π/(λ+0.5), with regard to their function values $D_{\lambda }\left(0\right)=\lambda +0.5$ and $D_{\lambda }\left(\pi /\left(\lambda +0.5\right)\right)=0$, respectively. We substitute the right-hand side of Equation (5) with $c=\left(\lambda \tau +1\right)A_{d}\left(\kappa \right)/\tau -1/\tau +0.5$ and obtain

$$\zeta_{0}=\frac{\pi \;(\lambda +0.5-c)}{{\left(\lambda +0.5\right)}^{2}}=\frac{\pi \left(\lambda +1/\tau \right)(1-A_{d}\left(\kappa \right))}{{\left(\lambda +0.5\right)}^{2}}\;.$$

In practice, the Newton’s method specified above with the proposed initialization results in convergence below the error threshold $10^{-7}$ within five steps, which is faster than our implementation in [37] by two orders of magnitude, thereby guaranteeing efficient sampling performance for online estimation. We now consider the following example to illustrate the efficacy of the proposed isotropic sampling scheme on von Mises–Fisher distributions of various configurations.

### 3.2. Example

**Figure 2:**
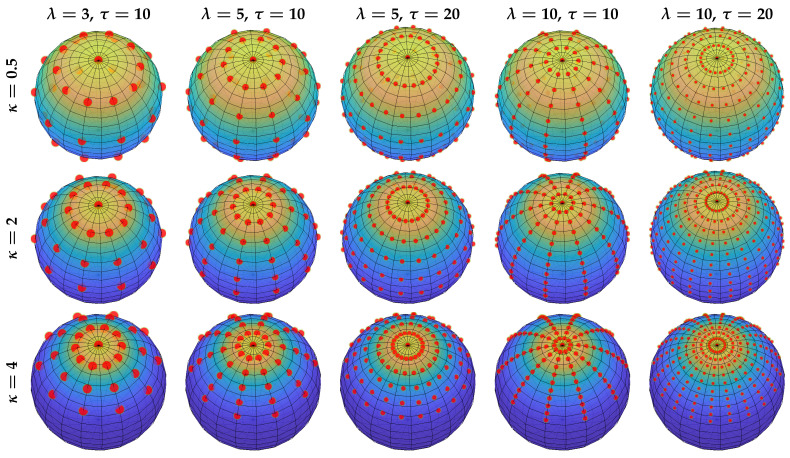
Illustration of the proposed isotropic deterministic sampling schemes with von Mises–Fisher distributions on $S^{2}$ of different parameterizations in Section 3.2. Samples (red dots) are uniformly weighted and dotted with sizes proportional to weights.

We parameterize the von Mises–Fisher distribution on the unit sphere $S^{2}$ with three concentration values κ={0.5,2,4}. Without loss of generality, the three distributions are given the same mode $\underline{\nu }={\left[\;0,0,1\;\right]}^{⊤}$. Five configurations are used for the proposed sampling method; i.e., (λ,τ)={(3,10),(5,10),(5,20),(10,10),(10,20)}. As shown in Figure 2, the isotropic sample sets are adapted to the dispersions for various parameterizations and configurations while preserving the mean resultant vector of the underlying distributions.

## 4. Progressive Unscented von Mises–Fisher Filtering

The proposed sampling method yields isotropic deterministic sample sets of arbitrary sizes that represent the underlying uncertainty more comprehensively for the unscented transform. As shown in our preceding work [37], the current unscented von Mises–Fisher filtering scheme is thus considerably enhanced for nonlinear estimation. However, for nonlinear and non-identity measurement models, the current paradigm simply reweights prior samples based on the likelihoods for the moment-matching of the posterior estimates. Although superior efficiency was shown over the approach using random samples, large sizes of deterministic samples are still desirable under strong nonlinearity [37] or with peaky likelihoods to avoid degeneration. To alleviate this issue, we propose the progressive unscented von Mises–Fisher filter using isotropic sample sets.

### 4.1. Task Formulation

We consider the following hyperspherical estimation scenario. The system model is assumed to be given as an equation of random variables:

$${\underline{x}}_{t+1}=\underline{a}\left({\underline{x}}_{t},{\underline{w}}_{t}\right)\;,$$

with ${\underline{x}}_{t},\;{\underline{x}}_{t+1}\in S^{d-1}\subset R^{d}$ representing the hyperspherical states and ${\underline{w}}_{t}\in W$ the system noise. The transition function $\underline{a}:S^{d-1}\times W\to S^{d-1}$ maps the state from time step *t* to t+1 under consideration of the noise term. The measurement model is given as

$${\underline{z}}_{t}=\underline{h}\left({\underline{x}}_{t},{\underline{v}}_{t}\right)\;,$$

where ${\underline{z}}_{t}\in Z,\;{\underline{v}}_{t}\in V$ are the measurement and the measurement noise, respectively. $\underline{h}:S^{d-1}\times V\to Z$ denotes the observation function.

### 4.2. Prediction Step for Nonlinear von Mises–Fisher Filtering

Given the setup above, one can use the Chapman–Kolmogorov equation to obtain the prior density from the last posterior $f_{t}^{e}\left({\underline{x}}_{t}\right)$:

(6) $$\begin{array}{cc} f_{t+1}^{p}\left({\underline{x}}_{t+1}\right)\; & =\int_{S^{d-1}}f_{t}^{e}\left({\underline{x}}_{t}\right)\;\int_{W}f\left({\underline{x}}_{t+1}\;|\;{\underline{w}}_{t},{\underline{x}}_{t}\right)\;f_{t}^{\underline{w}}\left({\underline{w}}_{t}\right)\;d{\underline{w}}_{t}\;d{\underline{x}}_{t}\;. \end{array}$$

We follow the generic framework of von Mises–Fisher filtering with samples facilitating the inference procedure. The estimates from the prediction and update steps are thus expressed in the form of von Mises–Fisher distributions. We allow arbitrary motion models. As explained in the following paragraphs, we use two different implementations of the prediction step according to the forms of the transition density.

(1) For a generic transition density, we first represent the posterior density $f_{t}^{e}\left({\underline{x}}_{t}\right)$ of the previous step using a sample set generated from the von Mises–Fisher distribution; namely,

(7) $$f_{t}^{e}\left({\underline{x}}_{t}\right)=\sum_{i=1}^{n}\omega_{t,i}^{e}\;\delta \left({\underline{x}}_{t}-{\underline{x}}_{t,i}^{e}\right)\;.$$

where δ(·) denotes the Dirac delta distribution and $\omega_{t,i}^{e}$ represents the sample weights satisfying $\sum_{i=1}^{n}\omega_{t,i}^{e}=1$. The prediction step in Equation (6) now turns into

(8) $$f_{t+1}^{p}\left({\underline{x}}_{t+1}\right)=\sum_{i=1}^{n}\omega_{t,i}^{e}\int_{W}f\left({\underline{x}}_{t+1}\;|\;{\underline{w}}_{t},{\underline{x}}_{t,i}^{e}\right)\;f_{t}^{\underline{w}}\left({\underline{w}}_{t}\right)\;d{\underline{w}}_{t}\;.$$

Similarly, the noise distribution $f_{t}^{\underline{w}}\left({\underline{w}}_{t}\right)$ of arbitrary form is also represented by a sample set; i.e., $f_{t}^{\underline{w}}\left({\underline{w}}_{t}\right)=\sum_{j=1}^{m}\omega_{t,j}^{\underline{w}}\;\delta \left({\underline{w}}_{t}-{\underline{w}}_{t,j}\right)$, with the sample weights $\sum_{j=1}^{n}\omega_{t,j}^{\underline{w}}=1$. Equation (6) is then reduced to

$$f_{t+1}^{p}\left({\underline{x}}_{t+1}\right)=\sum_{i=1}^{n}\sum_{j=1}^{m}\omega_{t,i}^{e}\cdot \omega_{t,j}^{\underline{w}}\cdot \delta \left({\underline{x}}_{t+1}-\underline{a}\left({\underline{x}}_{t,i}^{e},\;{\underline{w}}_{t,j}\right)\right)\;,$$

in which all elements of the Cartesian product of the posterior samples and the noise samples are propagated through the system function. As also shown in [37], the predicted von Mises–Fisher is fitted to the samples via moment matching.

(2) When the noise term ${\underline{w}}_{t}$ is additive and von Mises–Fisher-distributed, we obtain a transition density in the form of a von Mises–Fisher distribution [15]; namely,

$$f_{t}^{T}\left({\underline{x}}_{t+1}\;|\;{\underline{x}}_{t}\right)=f_{\mathrm{vMF}}\left({\underline{x}}_{t+1};\;{\underline{a}}_{t}\left({\underline{x}}_{t}\right),\kappa_{t}^{\underline{w}}\right)\;,$$

with $\underline{a}\left(t\right):S^{d-1}\to S^{d-1}$ being a noise-invariant system function of arbitrary form and $\kappa_{t}^{\underline{w}}$ denoting the concentration of the noise distribution. Then, the predicted density in Equation (6) can be expressed as

(9) $$\begin{array}{cc} f_{t+1}^{p}\left({\underline{x}}_{t+1}\right) & =\int_{S^{d-1}}f_{t}^{T}\left({\underline{x}}_{t+1}\;|\;{\underline{x}}_{t}\right)\;f_{t}^{e}\left({\underline{x}}_{t}\right)\;d{\underline{x}}_{t}\\ \; & =\int_{S^{d-1}}f_{\mathrm{vMF}}\left({\underline{x}}_{t+1};\;{\underline{a}}_{t}\left({\underline{x}}_{t}\right),\kappa_{t}^{\underline{w}}\right)\;f_{t}^{e}\left({\underline{x}}_{t}\right)\;d{\underline{x}}_{t}\;. \end{array}$$

To obtain the predicted density in Equation (9), we first propagate the posterior sample set ${\left\{{\underline{x}}_{t,i}^{e}\right\}}_{i=1}^{n}$ in Equation (7) through the motion model to obtain the propagated sample set ${\left\{{\underline{a}}_{t}\left({\underline{x}}_{t,i}^{e}\right)\right\}}_{i=1}^{n}$. To approximate the density after applying the system function, a von Mises–Fisher distribution $\mathrm{VMF}({\underline{\nu }}_{t},\kappa_{t})$ is then fitted to the propagated samples $\hat{\alpha }=\sum_{i=1}^{n}\omega_{t,i}\;{\underline{a}}_{t}\left({\underline{x}}_{t,i}^{e}\right)$ via moment matching as introduced in Equation (3). By convolving the fitted von Mises–Fisher distribution with that for the noise term, the predicted estimate $\mathrm{VMF}({\underline{\nu }}_{t+1}^{p},\kappa_{t+1}^{p})$ is obtained via ${\underline{\nu }}_{t+1}^{p}={\underline{\nu }}_{t}$ and $\kappa_{t+1}^{p}=A_{d}^{-1}\left(A_{d}\left(\kappa_{t}\right)A_{d}\left(\kappa_{t}^{\underline{w}}\right)\right)$. A detailed formulation of the method can be found in [15] (Algorithm 2).

### 4.3. Deterministic Progressive Update Using Isotropic Sample Sets

For nonlinear and non-identity measurement models, the posterior density can be obtained by reweighting the prior samples ${\left\{{\underline{x}}_{t,i}^{p}\right\}}_{i=1}^{n}$ using the likelihood $f_{t}^{L}\left({\hat{\underline{z}}}_{t}\;|\;{\underline{x}}_{t}\right)$ given the measurement ${\hat{\underline{z}}}_{t}$ as follows:

(10) $$f_{t}^{e}\left({\underline{x}}_{t}\;|\;{\hat{\underline{z}}}_{t}\right)\propto f_{t}^{L}\left({\hat{\underline{z}}}_{t}\;|\;{\underline{x}}_{t}\right)\;f_{t}^{p}\left({\underline{x}}_{t}\right)=\sum_{i=1}^{n}\omega_{i,t}^{p}\cdot f_{t}^{L}\left({\hat{\underline{z}}}_{t}\;|\;{\underline{x}}_{t,i}^{p}\right)\cdot \delta \left({\underline{x}}_{t}-{\underline{x}}_{t,i}^{p}\right)\;.$$

The posterior distribution is obtained by fitting a von Mises–Fisher distribution to the reweighted samples via moment matching. Directly applying the likelihood functions to the sample weights can be risky (regardless of whether they are generated randomly or deterministically) for strong nonlinearities or non-identity measurement models with peaky likelihoods due to the sample degeneration.

Therefore, we develop a novel update approach by deploying the proposed isotropic sampling to a progressive measurement update scheme [27]. More specifically, the likelihood in Equation (10) is decomposed into a product of *l* components:

(11) $$\begin{array}{cc} f_{t}^{e}\left({\underline{x}}_{t}\;|\;{\hat{\underline{z}}}_{t}\right) & \propto f_{t}^{L}\left({\hat{\underline{z}}}_{t}\;|\;{\underline{x}}_{t}\right)\cdot f_{t}^{p}\left({\underline{x}}_{t}\right)=\left(\prod_{k=1}^{l}{\left(f_{t}^{L}\left({\hat{\underline{z}}}_{t}\;|\;{\underline{x}}_{t}\right)\right)}^{\Delta_{k}}\right)\cdot f_{t}^{p}\left({\underline{x}}_{t}\right)\;, \end{array}$$

with $\sum_{k=1}^{l}\Delta_{k}=1$. The exponent $\Delta_{k}$ indicates the progression stride and is determined by a prespecified threshold ϵ∈(0,1] to bound the likelihood ratios among the deterministic samples according to

(12) $${\left(\frac{\min\left({\left\{v_{k,i}\right\}}_{i=1}^{\lambda \tau +1}\right)}{\max\left({\left\{v_{k,i}\right\}}_{i=1}^{\lambda \tau +1}\right)}\right)}^{\Delta_{k}}\geq \epsilon \;,\;\mathrm{with}\;v_{k,i}=f_{t}^{L}\left({\hat{\underline{z}}}_{t}\;|\;{\underline{x}}_{k,i}^{p}\right)$$

which is the likelihood of the prior isotropic sample ${\underline{x}}_{k,i}^{p}$ at the *k*-th progression step. Thus, we obtain

$$\Delta_{k}\leq \frac{\log(\epsilon )}{\log\left(v_{k}^{\min}\right)-\log\left(v_{k}^{\max}\right)}\;,$$

with $v_{k}^{\min}=\min\left({\left\{v_{k,i}\right\}}_{i=1}^{\lambda \tau +1}\right)$ and $v_{k}^{\max}=\max\left({\left\{v_{k,i}\right\}}_{i=1}^{\lambda \tau +1}\right)$, respectively. The progression stride is thus adaptively determined based on the variance of the samples’ likelihoods at the current progression step. We repeat this sampling–reweighting–fitting cycle based on the density obtained from the previous progression step until the likelihood is fully fused into the result (exponents $\Delta_{k}$ sum to one).

The procedure above is detailed with pseudo-code in Algorithm 2. We first initialize the posterior density with that obtained from the prediction step and set the remaining progression horizon to Δ=1 (Algorithm 2, line 1–2). At each progression step, an isotropic deterministic sample set is drawn from the current posterior density $f_{t}^{e}\left({\underline{x}}_{t}\right)$ (Algorithm 2, line 3–4). For each sample, we evaluate the likelihood for the measurement ${\underline{z}}_{t}$ and determine the maximal and minimal values of the likelihood values (Algorithm 2, line 5–7). Based on this, the current progression stride $\Delta_{k}$ is then computed according to Equation (12) (Algorithm 2, line 8). The posterior density is then fitted to the samples with the weights re-scaled according to the obtained progression stride $\Delta_{k}$, as shown in Equation (11) (Algorithm 2, line 9–10). We repeat the progression step until Δ reaches zero, which is when the entire likelihood has been incorporated into the density (Algorithm 2, line 10–11).
**Algorithm 2:** Isotropic Progressive Update
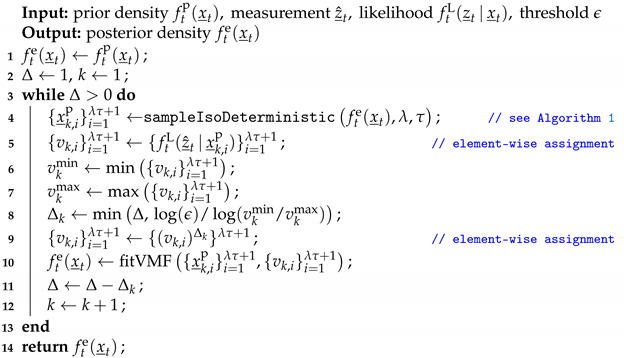


**Figure 3:**
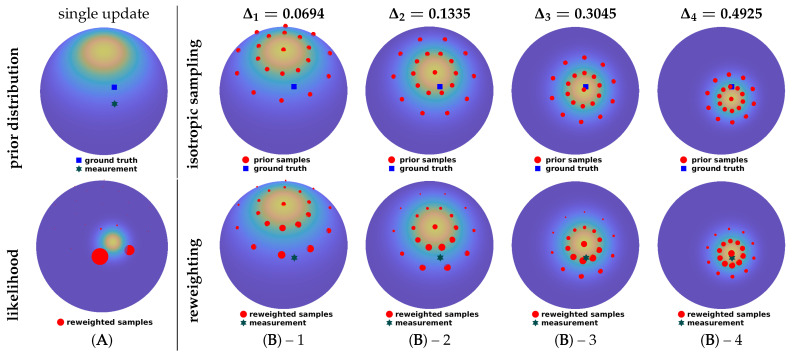
Illustration of the deterministic progressive update using isotropic sample sets. Sizes of red dots are proportional to their weights. The same isotropic sampling configuration, (λ,τ)=(2,10), is deployed for both the single-step and the progressive updates.

**Figure 4:**
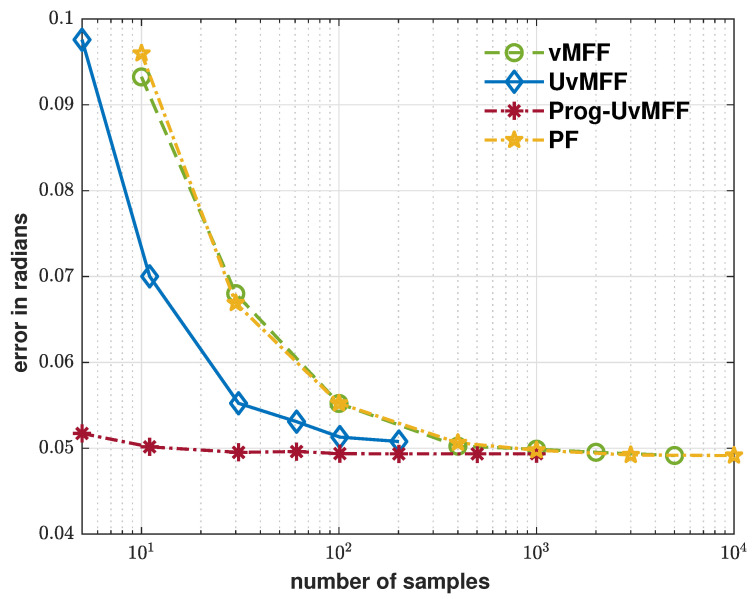
Error over sample numbers (log scale) for the evaluated filters. The configurations with five samples for UvMFF and Prog-UvMFF are based on the original UT-based sampling method in [15].

A full series of progression steps (for ϵ=0.02) using isotropic sample sets is illustrated in Figure 3 and compared with a conventional single-step update. For both approaches, 21 samples are used in the configuration (λ,τ)=(2,10). As shown in Figure 3A, given a prior von Mises–Fisher distribution on $S^{2}$ and a relatively peaky likelihood function, the single-step update deteriorates evidently due to sample degeneration. In contrast, the progressive approach (Figure 3B–1 to 4) performs four progression steps and achieves a superior fusion result.

## 5. Evaluation

We evaluate the proposed Prog-UvMFF using isotropic sample sets for nonlinear spherical estimation with a non-identity measurement model. To underline the merit of isotropic sampling and its integration into the proposed progressive deterministic update, we consider case 2 in Section 4.2 for the transition density with $f_{t}^{T}\left({\underline{x}}_{t+1}\;|\;{\underline{x}}_{t}\right)=f_{\mathrm{vMF}}\left({\underline{x}}_{t+1};\;{\underline{a}}_{t}\left({\underline{x}}_{t}\right),\kappa^{\underline{w}}\right)$, where ${\underline{x}}_{t},\;{\underline{x}}_{t+1}\in S^{2}$. The system dynamics is given as

$${\underline{a}}_{t}\left({\underline{x}}_{t}\right)=\frac{\sin\left(t/10\right)\cdot {\underline{x}}_{t}+\left(1-\sin(t/10)\right)\cdot \underline{\sigma }}{∥\sin\left(t/10\right)\cdot {\underline{x}}_{t}+\left(1-\sin(t/10)\right)\cdot \underline{\sigma }∥}\;,\;\mathrm{with}\;\underline{\sigma }={\left[\;1,1,1\;\right]}^{⊤}/\sqrt{3}\;,$$

which corresponds to the normalized linear interpolation [38] with a time-invariant interpolation ratio. We set the concentration κ of the von Mises–Fisher-distributed transition density to 50. Unlike the evaluation scenario in [37], the posterior of the previous step is propagated using samples and the predicted von Mises–Fisher prior is obtained by convolving the fitted density with the system noise as introduced in the second case of Section 4.2.

The nonlinear and non-identity measurement model yields the spherical coordinates (azimuth and elevation) of the state ${\underline{x}}_{t}={\left[\;{\underline{x}}_{t,1},{\underline{x}}_{t,2},{\underline{x}}_{t,3}\;\right]}^{⊤}$, i.e.,

$${\underline{z}}_{t}=\underline{h}\left({\underline{x}}_{t}\right)+{\underline{v}}_{t}\;,\;\mathrm{with}\;\underline{h}\left({\underline{x}}_{t}\right)={\left[\begin{array}{c} \;\arctan\left(\frac{{\underline{x}}_{t,2}}{{\underline{x}}_{t,1}}\right),\;\arctan\left(\frac{{\underline{x}}_{t,3}}{\sqrt{{\underline{x}}_{t,1}^{2}+{\underline{x}}_{t,2}^{2}}}\right)\; \end{array}\right]}^{⊤}.$$

The additive measurement noise is zero-mean Gaussian-distributed; namely, ${\underline{v}}_{t}∼N\left(\underline{0},\Sigma^{\underline{v}}\right)$, with $\underline{0}\in R^{2}$ and covariance $\Sigma^{\underline{v}}\in R^{2\times 2}$. Thus, the likelihood function is

(13) $$f_{t}^{L}\left({\underline{z}}_{t}\;|\;{\underline{x}}_{t}\right)=f_{N}\left({\underline{z}}_{t}-\underline{h}\left({\underline{x}}_{t}\right)\right)\;.$$

We set the covariance $\Sigma^{\underline{v}}=0.002\cdot I_{2\times 2}$ to induce a peaky likelihood function.

Three variants from the von Mises–Fisher filtering framework are considered for the evaluation: the plain von Mises–Fisher filter (vMFF) based on random sampling, the unscented von Mises–Fisher filter (UvMFF) using deterministic sample sets and the progressive UvMFF (Prog-UvMFF), which fuses the measurements via progressions. The threshold ϵ controlling the progression stride in Equation (12) is set to 0.02. The random samples in the vMFF are drawn using the approach in [13]. To generate deterministic samples in the UvMFF and the Prog-UvMFF, we involve both of the UT-based methods with a fixed sample size (as for $S^{2}$, n=5) [15] and the proposed isotropic sampling with configurable sizes. Furthermore, we run the particle filter (PF) with a typical sampling–importance resampling approach as a baseline. All the filters are initialized using the same prior von Mises–Fisher distribution $\mathrm{VMF}({\underline{\nu }}_{0},\kappa_{0})$, where ${\underline{\nu }}_{0}={\left[\;0,0,1\;\right]}^{⊤}$ and $\kappa_{0}=50$. The error between the ground truth $\underline{x}$ and the estimated state $\hat{\underline{x}}$ is quantified by the arc length on $S^{2}$ in radians; i.e.,

$$E\left(\underline{x},\hat{\underline{x}}\right)=\mathrm{acos}\left({\underline{x}}^{⊤}\hat{\underline{x}}\right)\;.$$

The scenario is simulated for 30 time steps in each run. A total of 1000 Monte Carlo runs is used for the evaluation. A broad scale of sample sizes (from 5 to $10^{4}$) is considered. Deviations are summarized in the form of the root mean squared error (RMSE).

**Figure 5:**
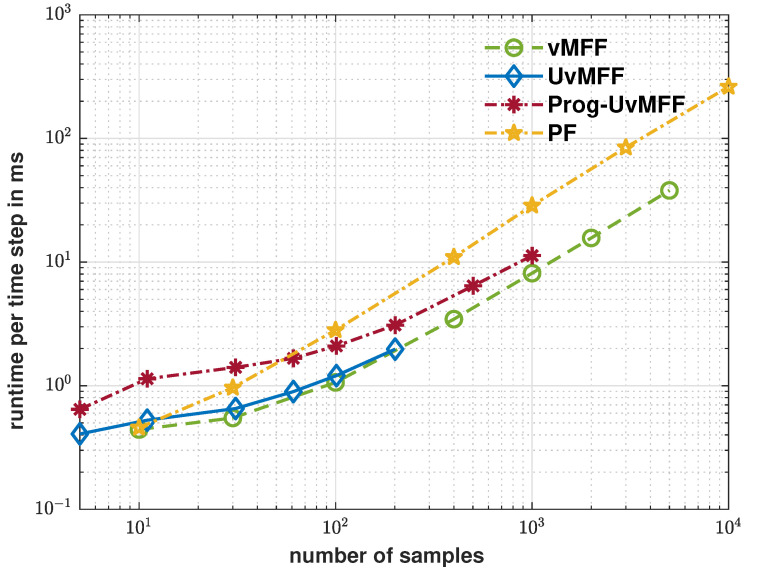
Runtime for each time step in ms over sample size for the evaluated filters.

**Figure 6:**
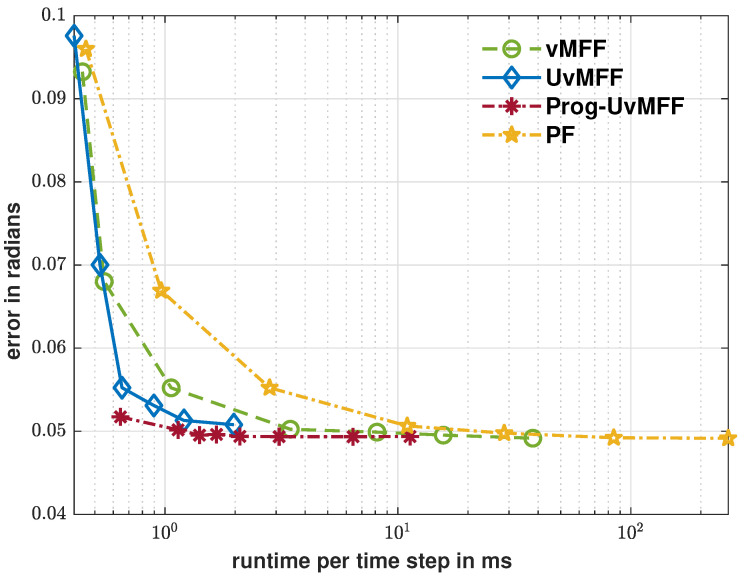
Error over runtime for the evaluated filters.

The evaluation results are plotted in Figure 4, Figure 5 and Figure 6. As shown by the blue curve in Figure 4, the proposed isotropic sampling method allows the ordinary UvMFF [15] to deploy configurable sizes (any number larger than five) of deterministic samples, thereby achieving a superior performance over the random sampling-based filters (vMFF and PF). Due to the peaky likelihood function in Equation (13), however, its progressive variant (Prog-vMFF) delivers much better tracking accuracy (with the same sample size) as well as convergence. Figure 5 shows the runtime efficiency of the evaluated filters. As indicated by the green and blue curves, the runtime of the proposed isotropic sampling method is similar to that of the random one (as the two filters are based on the same filtering procedure) and the two filters are faster than the PF with the same numbers of samples. For the proposed Prog-UvMFF, the progressive measurement fusion induces slightly more runtime than the fusion with a conventional single-step update (while still being faster than the PF). The cost-efficiency (in terms of runtime) of different filters is displayed in Figure 6. Given the same amount of processing time, the proposed isotropic sampling method facilitates the UvMFF in delivering less error than the random counterpart. Furthermore, it enables the Prog-UvMFF to achieve the best tracking accuracy in conjunction with the progressive update.

## 6. Conclusions

In this work, we propose a new deterministic sampling method for generating equally weighted sample sets of configurable sizes from von Mises–Fisher distributions in arbitrary dimensions. Based on hyperspherical geometries, the sample sets are placed in isotropic layouts adapted to the dispersion of the underlying distribution while satisfying the requirement of the unscented transform. To further enhance nonlinear von Mises–Fisher filtering techniques, we propose a deterministic progressive update step to handle non-identity measurement models. The final product, the Prog-UvMFF, is built upon the progressive filtering scheme with isotropic sample sets and delivers evidently superior performance over state-of-the-art von Mises–Fisher filters and the PF for nonlinear hyperspherical estimation.

Besides the theoretical contribution to recursive estimation for directional manifolds, the presented progressive unscented von Mises–Fisher filter supports generic measurement models that are directly derived from the true sensor modalities. Thus, it is also of interest to evaluate the filter’s performance in real-world tasks. Potential application scenarios include orientation estimation using omnidirectional vision [5], visual tracking on unit hyperspheres [39], bearing-only localization in sensor networks [40], wavefront orientation estimation in the surveillance field [29] and sound source localization [41].

There are multiple directions for further research. In addition to only matching the mean resultant vector, the higher-order shape information of a von Mises–Fisher distribution can be considered, which may lead to further enhancements in the filter performance. For this, deterministic samples can be non-uniformly weighted. Since hyperspherical uncertainties can be of an arbitrary shape in practice, parametric filtering can be error-prone in certain cases (e.g., in the presence of multimodality). Mixtures of von Mises–Fisher distributions can be exploited for more exact modeling, and corresponding recursive estimators are promising.
